# Association between Training Load and Well-Being Measures in Young Soccer Players during a Season

**DOI:** 10.3390/ijerph18094451

**Published:** 2021-04-22

**Authors:** Hadi Nobari, Ana Ruivo Alves, Hamed Haghighi, Filipe Manuel Clemente, Jorge Carlos-Vivas, Jorge Pérez-Gómez, Luca Paolo Ardigò

**Affiliations:** 1 Department of Physical Education and Sports, University of Granada, 18010 Granada, Spain; 2 Sports Scientist, Sepahan Football Club, Isfahan 81887-78473, Iran; 3 Department of Exercise Physiology, Faculty of Sport Sciences, University of Isfahan, Isfahan 81746-7344, Iran; 4 HEME Research Group, Faculty of Sport Sciences, University of Extremadura, 10003 Cáceres, Spain; jorge.carlosvivas@gmail.com; 5 Department of Arts, Humanities and Sports, Polytechnic Institute of Beja, School of Education, 7800-295 Beja, Portugal; ana.alves@ipbeja.pt; 6 Research Center in Sports Sciences, Health Sciences and Human Development, CIDESD, 5001-801Vila Real, Portugal; 7 Laboratory of Physical Activity and Health, Polytechnic Institute of Beja, School of Education, 7800-295 Beja, Portugal; 8 Department of Sport Injuries and Corrective Exercises, Faculty of Sport Sciences, University of Isfahan, Isfahan 81746-7344, Iran; hamed8haghighi@gmail.com; 9 Polytechnic Institute of Viana do Castelo, School of Sport and Leisure, 4900-347 Viana do Castelo, Portugal; Filipe.clemente5@gmail.com; 10 Institute of Telecommunications, IT-Branch Covilhã, 6200-001 Covilhã, Portugal; 11 Department of Neurosciences, Biomedicine and Movement Sciences, School of Exercise and Sport Science, University of Verona, 37134 Verona, Italy; luca.ardigo@univr.it

**Keywords:** athlete monitoring, fatigue, football, performance, psychological, soreness, sports training, team sports, young

## Abstract

This study aimed to analyze the correlations among weekly (w) acute workload (wAW), chronic workload (wCW), acute/chronic workload ratio (wACWR), training monotony (wTM), training strain (wTS), sleep quality (wSleep), delayed onset muscle soreness (wDOMS), fatigue (wFatigue), stress (wStress), and Hooper index (wHI) in pre-, early, mid-, and end-of-season. Twenty-one elite soccer players (age: 16.1 ± 0.2 years) were monitored weekly on training load and well-being for 36 weeks. Higher variability in wAW (39.2%), wFatigue (84.4%), wStress (174.3%), and wHI (76.3%) at the end-of-season were reported. At mid-season, higher variations in wSleep (59.8%), TM (57.6%), and TS (111.1%) were observed. Moderate to very large correlations wAW with wDOMS (r = 0.617, *p* = 0.007), wFatigue, wStress, and wHI were presented. Similarly, wCW reported a meaningful large association with wDOMS (r = 0.526, *p* < 0.001); moderate to very large associations with wFatigue (r = 0.649, *p* = 0.005), wStress, and wHI. Moreover, wTM presented a large correlation with wSleep (r = 0.515, *p* < 0.001); and a negatively small association with wStress (r = −0.426, *p* = 0.003). wTS showed a small to large correlation with wSleep (r = 0.400, *p* = 0.005) and wHI; also, a large correlation with wDOMS (r = 0.556, *p* = 0.028) and a moderate correlation with wFatigue (r = 0.343, *p* = 0.017). Wellness status may be considered a useful tool to provide determinant elite players’ information to coaches and to identify important variations in training responses.

## 1. Introduction

In soccer, components such as anaerobic power, aerobic fitness, agility, speed, and flexibility have been considered crucial to acquire and maintain over the season [1]. To develop soccer players’ physical fitness, the training process should consider the individual athletes’ needs, including a proper implementation of frequency, volume, and intensity (i.e., training load) in all training sessions [2]. To the development of training process in young soccer players, a careful monitorization of training load seems to be relevant. Furthermore, a suitable training load is fundamental for short-term performance development and to empower young athletes [3]. In order to complement the training load monitorization and to avoid unbalance in athletic performance, a special consideration should also be applied on well-being variables [4]. It is established that different stress factors (e.g., anxiety, fatigue) could negatively influence the performance and outcomes during the season [5]. Moreover, factors such as fitness level, mood status, session’s intensity, accumulate load of training, and specific moments of the season (e.g., competition) may produce distinct perceptions of load throughout the season [6,7]. In this sense, the relationship between players’ wellness (where well-being raters can be assessed based on a self-report questionnaire relative to sleep quality, delayed onset muscle soreness (DOMS), fatigue, and stress) and training load (e.g., acute and chronic workload) has received a growing interest in recent years. The literature provides significant interactions between DOMS, stress, fatigue perception, and sleep quality [8,9,10]. For instance, previous studies which focused on wellness and load in soccer players reported an association between both components during a specific competition and even across the season. Moreover, specific wellness factors such as neuromuscular fatigue and soreness can be sensitive to overall load and load changes [11,12,13]. Additionally, the accumulated perceived exertion has been reported to have a powerful association with aerobic power (specifically, with the improvement in the highest speed accomplished during 30–15 Intermittent Fitness Test (30–15_IFT_)) of professional soccer players [14]. However, there is a lack of investigation assessing training load and well-being in youth soccer players over the season. To the best of our knowledge, only one article shows the importance of training load in the wellness management of elite junior players, and its possibility to reduce the risk of injuries [15]. Even though the aforementioned research of Lathlean et al. [15] has evidence to the literature, there is a paucity of studies which analyze the relationship between young players’ perceived well-being with acute workload (AW) and chronic workload (CW) variables. Only one study has been conducted in this area [16]. Nobari et al. [16] aimed to analyze the associations between training load and wellbeing status of soccer players, reporting a large association between acute training load and well-being indicators (i.e., fatigue, stress, sleep, Hooper index, and DOMS). Knowing these associations may provide helpful information for sports scientists, sport coaches, or even strength and conditioning coaches to effectively manage the training process, acquire improvements, and prevent poor adaptations, which can interfere in sleep quality, stress, and DOMS, and thereby impair performance [17]. Furthermore, the analysis of both perceived load and wellness status is also simple to obtain and has a low-cost [18]. In this sense, the training load (e.g., AW and CW), training strain, and monotony for perceived load may provide valuable information for coaches and practitioners to better understand weekly session distribution and workload effects. Wellbeing indicators may offer relevant evidence to coaches in order to adapt training sessions, to reduce fatigue, and to potentiate soccer players’ performance. Therefore, this study aimed to analyze the correlations among the weekly (w) acute workload (wAW), chronic workload (wCW), acute: chronic workload ratio (wACWR), training monotony (wTM), training strain (wTS), sleep quality (wSleep), wDOMS, fatigue (wFatigue), stress (wStress), and Hooper index (wHI) of young soccer players in different periods of the season (pre-, early, mid-, and end-of-season). We hypothesized that well-being status could be highly related with training load overall the season.

## 2. Materials and Methods

### 2.1. Participants

Twenty-one elite young soccer players participated in this study (mean ± standard deviation (SD); age, 16.1 ± 0.2 years; height, 176.8 ± 5.6 cm; body mass, 67.3 ± 5.7 kg; BMI, 21.5 ± 1.4 kg/m^2^; VO_2max_, 47.6 ± 3.8 mL·kg^−1^·min^−1^). The participants were the main players of an elite soccer team under-17 (U17) in the 2019–2020 season. Inclusion criteria in this study were: (i) players who participated in at least 90% of training seasons; (ii) players could not participate any additional training outside the specific team training during this study; (iii) each player who did not participate in the match during a week performed a separate training session without the ball or in small side games. This was done to match the number of practice sessions during the season.

### 2.2. Sample Size

Studies have shown high to very high correlations between training load and well-being in youth soccer players [16,19,20]. With this background, the sample size was analyzed to obtain a suitable number population with at least 80% statistical power. The variables applied to calculate the statistical power were: two-tailed, α error of <0.05, and high effect size (0.55). Twenty-one players were needed to reach 82% power.

### 2.3. Experimental Approach to the Problem

This study is a descriptive-longitudinal for the monitored full-season for an elite soccer team. Daily monitoring was observed by players for 36 weeks (W) from the beginning of the preparation season. The full-season was divided into four periods according to the team competition schedule: Pre-season, W1 to W5; Early season, W6 to W13; Mid-season, W14 to W31; and End-of-season, W32 to W36 (Table 1) to analyze the relationship between workload parameters and well-being categories across the full-season. Players trained at least three times per week during the season. Players had experience, at least 3 years, of providing self-report for their rating of perceived exertion (RPE) and HI on a daily basis.

They reported, individually, the wellness status and RPE 30 min before and after the training and competition session. The researcher of this study, who was the strength and conditioning coach of the team, asked the question and recorded the information. Finally, multiplying the training time per minute in session-RPE (score in CR-10 scale), the daily training load was obtained [16,21]. For example, the training time was 110 min and the RPE score announced by the player was 4. The training load of that player was equal to 440 arbitrary units (A.U). These data were used to obtain workload information and analysis of AW, CW, ACWR, TM, and TS [21,22]. The CW and ACWR were calculated from the uncoupled formula [23]; therefore, these variables were reported from the third and fourth weeks, respectively. The 30–15_IFT_ was performed, and the special formula of this test was used to calculate the maximum oxygen consumption (VO_2max_) of the players [24].

### 2.4. Anthropometric Measures

Anthropometric variables such as standing height (Seca model 213, Germany with an accuracy of ±5 mm) and weight (Seca model 813, the UK with an accuracy of 0.1 per kg) were measured using the techniques of the international society for the advancement of kinanthropometry [25,26]. A person with more than 5 years of experience in anthropometric research performed the measurements [27,28]. The standing height and weight were measured once in the pre-season between 08:00–11:00 AM [27].

### 2.5. Aerobic Power Test

This test was performed to compute the subjects′ VO_2max_ [24]. The 30–15_IFT_ includes running for 30 s and walking for 15 s. Which is done as a shuttle between three lines with a distance of 20 m (i.e., two 20 m). Players started running from line A to line B and C and then returned to the same path until the 30-s beep was announced. The first stage started with 8 km/h^−1^ and each stage increased its speed by 0.5 km/h^−1^. The 30–15_IFT_ continued until the subjects could not continue the test or the two-meter lines were not reached three times in a row. The subjects were encouraged to perform at their maximum performance during the test. To estimate the VO_2max_ of the players, the 30–15_IFT_ was performed and afterward was calculated by the following formula: VO_2max_ (ml.kg^−1^·min^−1^) = 28.3 − (2.15 × 1) − (0.741 × 17 yrs.) − (0.0357 × Weight) + (0.0586 × 17 yrs. × VIFT) + (1.03 × VIFT). VIFT was considered as the end of the player′s final speed in the test exhaustion.

### 2.6. Monitoring Internal Training Loads

Prior to the research, players were familiarized with the scale (at least three years using RPE). Players were monitored daily by the RPE using the CR-10 Borg’s scale [21]. In previous studies, the validity and reliability of this scale has been confirmed to determine the training load of athletes [22]. The question was “How intense was your session?”. Players answered this question 30 min after the end of training or match session [16,20,26]. This time was calculated exactly when players finished training or matches, then the question was asked. The training sessions time (in minutes) were recorded [26,29]. The RPE of each session was calculated multiplying the score in CR-10 scale by the duration of the session in minutes, as a measure of internal load.

### 2.7. Calculate Training Load

In this study, parameters workloads were calculated as follows [22]: (i) wAW for daily workloads was total sessions that held on per week; (ii) wCW, the average accumulated data of the previous 3-weeks, using the uncoupled formula [16,30]; (iii) also, to reduce the reporting error, the uncoupled formula was used to calculate the ACWR. For example, ACWR4 = [wAL 4)/(0.333 × (wAL 2 + 3 + 4)]; (iv) wTM, the relation of average of the wAW by SD during 1-week; (v) wTS, is the multiplication of wAW by wTM.

### 2.8. Wellbeing Status Monitoring

HI is a self-report questionnaire, which is based on a 7-point scale involving the well-being status relative to stress, fatigue, DOMS, and sleep quality [7,31,32]. The HI is the summation of the four subjective ratings. The HI was applied 30-min before each session [33]. In this questionnaire, the number one means good condition and number seven means very bad condition. Players had to answer the above four variables, based on their mental feelings (self-report) before each training session. However, for muscle DOMS, players had to contract the thigh muscle in its range of motion, and then announce their desired number based on the pain they felt. All questions and answers were done individually. Prior to the research, players were familiarized with the scale (at least three years using HI). The following accumulated data were obtained for each variable by the sum during the week: wStress, wFatigue, wDOMS, wSleep, and wHI.

### 2.9. Statistical Analysis

The Statistical Package for the Social Sciences (SPSS, version 25.0; IBM SPSS Inc., Chicago, IL, USA) was used for computations. Descriptive statistics are presented as mean and SD. Additionally, the weekly changes and coefficients of variation are shown as percentage. Data normality and homogeneity were checked applying the Shapiro–Wilks and Levene’s tests, respectively. After that, the associations between training workload measures and well-being variables were made by using a repeated measures correlation test ®. Correlation’s thresholds were defined as follow [34]: ≤0.1, trivial; >0.1 to ≤0.3, small; >0.3 to ≤0.5, moderate; >0.5 to ≤0.7, large; >0.7 to ≤0.9, very large; and ≥0.9, nearly perfect. The correlations were always presented with 95% confidence interval (CI). The alpha level was set at *p* ≤ 0.05. We performed an a priori estimation of power and sample size through the statistical software (G-Power; University of Dusseldorf, Dusseldorf, Germany).

## 3. Results

**Figure 1:**
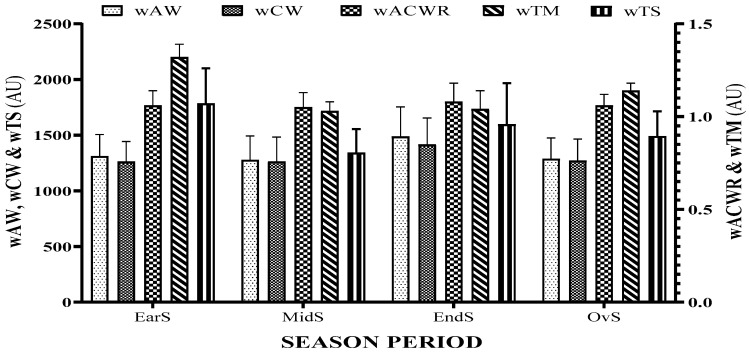
Summary of every season period for training load variables: weekly acute workload (wAW), weekly chronic workload (wCW), weekly acute/chronic workload ratio (wACWR), weekly training monotony (wTM), and weekly training strain (wTS).

**Figure 2:**
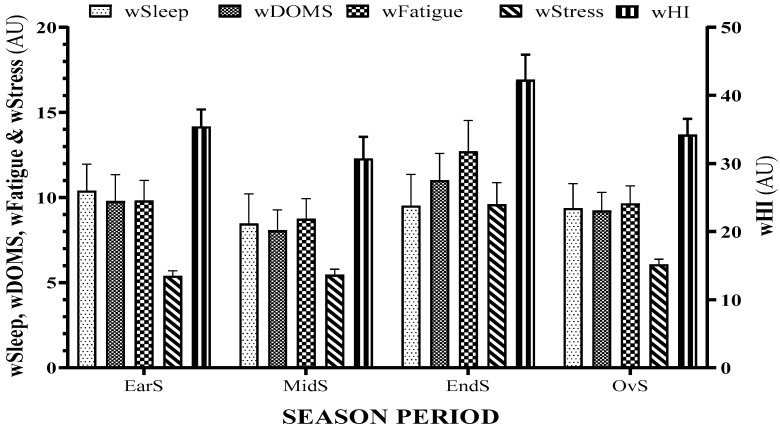
Summary of every season period for well-being variables: weekly sleep quality (wSleep), weekly delayed onset muscle soreness (wDOMS), weekly fatigue (wFatigue), weekly stress (wStress), and weekly Hooper index (wHI).

Figure 1 and Figure 2 show the summary of every season period for training load variables (wAW, wCW, wACWR, wTM, and wTS) and well-being variables (wSleep, wDOMS, wFatigue, wStress, and wHI), respectively.

**Figure 3:**
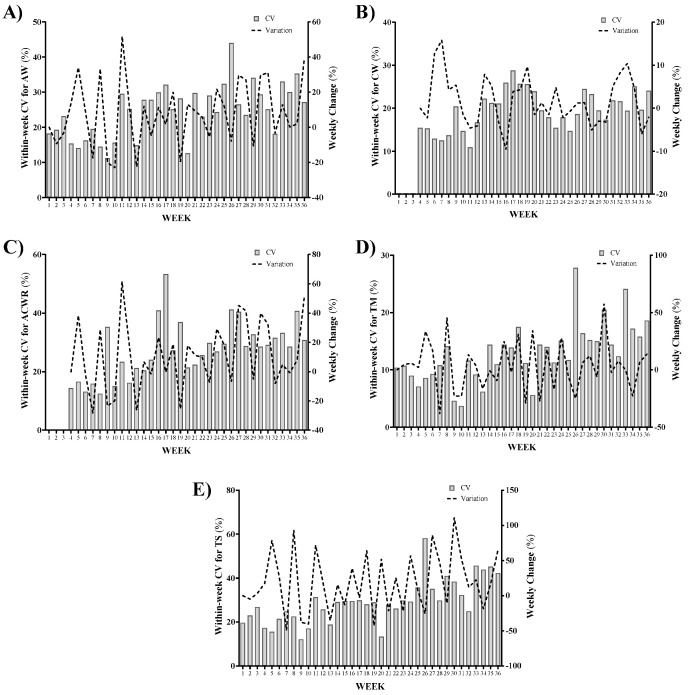
Within-week coefficient of variations (CV) and between-week variations (%) for (**A**) acute workload (AW), (**B**) chronic workload (CW), (**C**) weekly acute/chronic workload ratio (wACWR), (**D**) weekly training monotony (wTM), and (**E**) weekly training strain (wTS) across the season.

Within-week coefficient of variations (CV) and between-week variations (%) for AW, CW, wACWR, wTM, and wTS across the season are displayed in Figure 3. The highest wAW variation occurred from week 35 to week 36 (39.2%); while the highest reduction (−23.0%) was observed from week 9 to week 10 (Figure 3A). The greatest CV happened in week 26 (44%) (Figure 3A). The highest wCW variation was observed from week 6 to week 7 (15.8%) and the maximum decrease (−9.6%) occurred from week 15 to week 16 (Figure 3B). Regarding wACWR, the highest increase and reduction were 61.5% (from week 10 to week 11) and −28.3% (from week 6 to week 7), respectively (Figure 3C). Coincidently, the greatest within-week variation happened in week 17 for wCW (28.8%) and wACWR (53.3%) (Figure 3B,C). Likewise, both wTM and wTS presented the highest increase from week 29 to week 30 (wTM: 57.6%; wTS: 111.1%), and the maximum decrease from week 6 to week 7 (wTM: 57.6%; wTS: 111.1%) (Figure 3D,E). Moreover, both wTM and wTS presented the greatest within-week variation (wTM: 27.8%; wTS: 58.2%) in week 26 (Figure 3,E).

**Figure 4:**
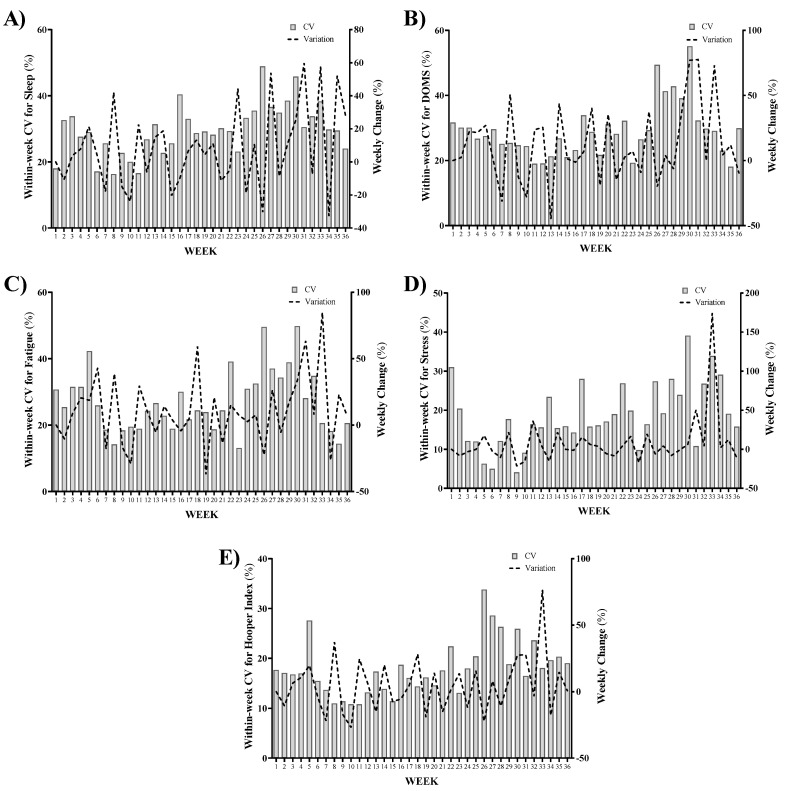
Within-week coefficient of variations (CV) and between-week variations (%) for (**A**) weekly sleep quality (wSleep), (**B**) weekly delayed onset muscle soreness (wDOMS), (**C**) weekly fatigue (wFatigue), (**D**) weekly stress (wStress), and (**E**) weekly Hooper index (wHI) across the season.

Figure 4 shows the within-week CV and between-week variations (%) for well-being categories: wSleep, wDOMS, wFatigue, wStress, and wHI across the season. The highest wSleep variation occurred from week 30 to week 31 (59.8%); while the highest reduction (−32.7%) was observed from week 33 to week 34 (Figure 4A). The greatest within-week variation happened in week 26 (48.9%) (Figure 4A). The highest wDOMS variation was observed from week 30 to week 31 (77.6%) and the maximum decrease (44.7%) occurred from week 12 to week 13 (Figure 4B). Regarding wFatigue, the highest increase and reduction were 84.8% (from week 32 to week 33) and −37.1% (from week 18 to week 19), respectively; and the highest CV occurred in week 30 (49.8%) (Figure 4C). Like wFatigue, the highest increment of wStress happened from week 32 to 33 (174.3%) and the greatest CV was in week 30 (39.1%); while the most important decrease in wStress occurred from week 8 to 9 (−21.5%) (Figure 4D). Finally, the highest increment and reduction in wHI were 76.3% (from week 32 to 33) and −26.9% (from week 9 to 10), respectively. The greatest CV for wHI was presented in week 26 (33.8%) (Figure 4E).

**Figure 5:**
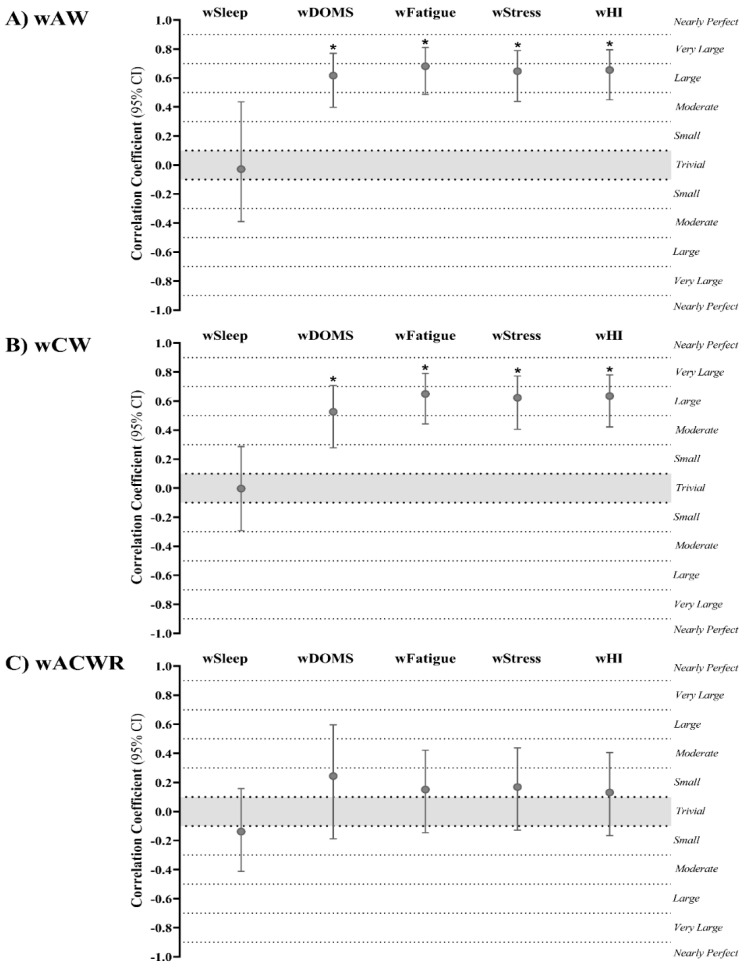
Repeated measures correlation outcomes (95% CI) of (**A**) weekly acute workload (wAW), (**B**) weekly chronic workload (wCW), and (**C**) weekly acute/chronic workload ratio (wACWR) with well-being categories. wSleep, weekly sleep; wDOMS, weekly delayed onset muscle soreness; wFatigue, weekly fatigue; wStress, weekly stress; wHI, weekly Hooper index. * Correlation coefficient is significant at *p*-value ≤ 0.05.

**Figure 6:**
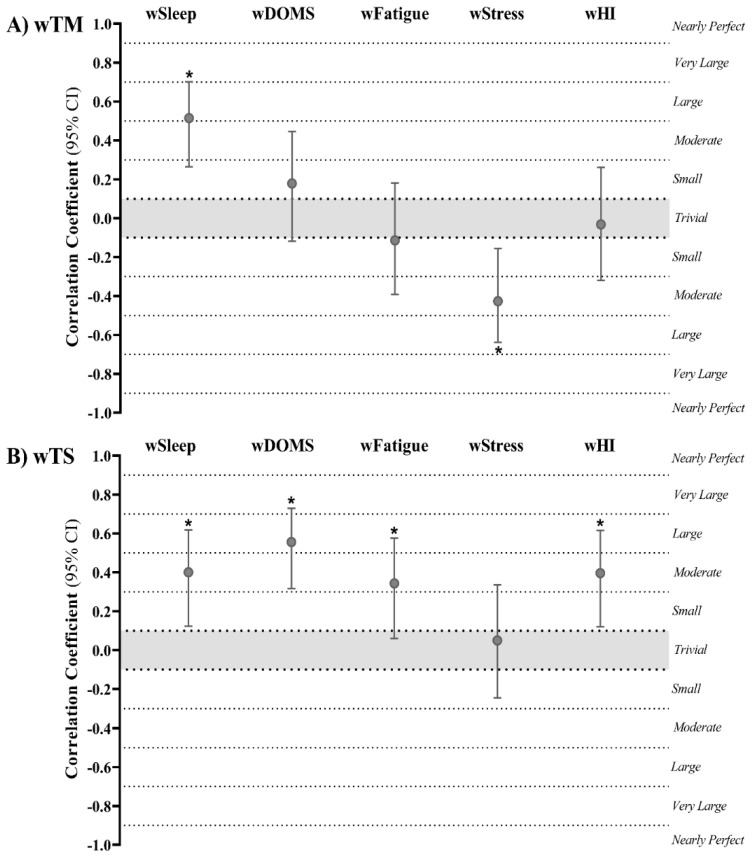
Repeated measures correlation outcomes (95% CI) of (**A**) weekly training monotony (wTM) and (**B**) weekly training strain (wTS) with well-being categories. wSleep, weekly sleep; wDOMS, weekly delayed onset muscle soreness; wFatigue, weekly fatigue; wStress, weekly stress; wHI, weekly Hooper index. * Correlation coefficient is significant at *p*-value ≤ 0.05.

Repeated measures correlation outcomes between workload parameters and well-being categories are presented in Figure 5 and Figure 6. Results showed moderate to very large correlations of wAW with wDOMS (r = 0.617; 95% CI: 0.398 to 0.770; *p* = 0.007), wFatigue (r = 0.681; 95% CI: 0.487 to 0.811; *p* = 0.003), wStress (r = 0.647; 95% CI: 0.439 to 0.789; *p* = 0.006), and wHI (r = 0.655; 95% CI: 0.450 to 0.794; *p* = 0.004) (Figure 5A). Similarly, wCW presented a meaningful small to very large association with wDOMS (r = 0.526; 95% CI: 0.278 to 0.708; *p* < 0.001) and significant moderate to very large associations with wFatigue (r = 0.649; 95% CI: 0.443 to 0.791; *p* = 0.005), wStress (r = 0.623; 95% CI: 0.406 to 0.773; *p* = 0.006), and wHI (r = 0.635; 95% CI: 0.422 to 0.781; *p* = 0.003) (Figure 5B). Contrarily, wACWR was not correlated with any well-being category (Figure 5C).

Regarding wTM and wTS, the outcomes were slightly different. Specifically, wTM only presented a significant small to very large correlation with wSleep (r = 0.515; 95% CI: 0.264 to 0.701; *p* < 0.001) and negative small to large association with wStress (r = −0.426; 95% CI: −0.638 to −0.155; *p* = 0.003) (Figure 6A). However, wTS showed a meaningful small to large correlation with wSleep (r = 0.400; 95% CI: 0.124 to 0.618; *p* = 0.005) and wHI (r = 0.396; 95% CI: 0.120 to 0.616; *p* = 0.005); a moderate to very large significant association with wDOMS (r = 0.556; 95% CI: 0.317 to 0.729; *p* = 0.028); and a meaningful trivial to large correlation with wFatigue (r = 0.343; 95% CI: 0.059 to 0.576; *p* = 0.017) (Figure 6).

## 4. Discussion

The purpose of this study was to analyze the correlations among the wAW, wCW, wACWR, wTM, wTS, wSleep, wDOMS, wFatigue, wStress, and wHI of young elite soccer players in different periods of the season (pre-, early, mid-, and end-of-season). Higher variation of wAW were observed in the end-of-season, which also coincided with higher variation of wFatigue, wStress, and wHI during the end-of-season. The highest reduction of the wAW was obtained in the early season as well as the highest reduction values of wDOMS, wStress, and wHI during also the early season. Regarding to the wCW, the most significant variation was showed in the early season; however, the most significant reduction was observed at mid-season being compatible with higher reduction values on wFatigue. Interestingly, with regard to the wACWR, the most substantial increase was in the early season, and also the highest reduction occurred in the same period with congruent results in highest reduction on the wTM, wTS, wDOMS, wStress, and wHI. Finally, for both wTM and wTS, a higher variation in mid-season was observed, with consistent results on wSleep and wDOMS in the same period. In this sense, more variability in wAW that occurred in the end-of-season was harmonious with higher variability of fatigue, stress, and HI. This increase on well-being status was possibly caused by the participation of the team in the important competition games of the season, which may have a higher significance for players, creating an anticipatory effect associated with these decisive matches and related anxiety, with less efficient time for recovery [35].

Concerning the results of correlations, wAW presented moderate to very large correlations with wDOMS, with wFatigue, with wStress, and with wHI. Similarly, wCW reported meaningful small to very large associations with wDOMS and significant moderate to very large associations with wFatigue, with wStress, and with wHI. Curiously, wACWR did not showed correlation with any well-being category. On the other hand, wTM presented a significant small to very large correlation with wSleep and a negatively small to large association with wStress. Finally, wTS showed a meaningful small to large correlation with wSleep and wHI, a moderate to very large correlation with wDOMS, and a meaningful trivial to large correlation with wFatigue. In fact, these results were also reported in a previous systematic review that evaluated perceived stress and its symptoms, showing that both categories were sensitive to acute changes in load [36]. This tendency was also revealed in the study of Lathlean and colleagues [15], which concluded that a higher variation in load with lowered monotony is associated to a higher soreness. Regarding both wCW and wAW, if the lower variability observed in the mid-season is considered, the highest variation of wSleep obtained in the same period of the season can be explained by the fact that sleep perception is sensitive to the workload. These facts were also consistent with previous studies [37,38]. Moalla et al. [37] studied the relationship between daily training load and perceived wellness characteristics of professional soccer players, reporting that the perceived sleep is moderately related to the daily training load. Clemente et al. [38], by aiming to study the relationship between perceived internal load and wellness status of elite male volleyball players, found a very large correlation between perceived sleep and weekly acute load.

Controlling the training load of soccer players (e.g., AW and CW) is essential to guarantee a short-term performance development and enables the athletes’ futures [3]. In this respect, the suitable monitorization of training load will also be important to development and fundamental movement skills, contributing to optimize the athlete performance, and diminish the injury rate [39]. Critical reports have emerged on the ACWR through the controversy of findings in the literature (positive and negative associations) exposed when injury rate in athletes is assumed [40,41]. However, there are no doubts regarding the pertinence of control the ACWR to monitor changes in fitness levels of players [42], to monitor changes in wellness status of the players [43], and to determine the optimal weekly load division to ensure the sufficient post-match recovery and prevent pre-match fatigue [44].

Following our results, in the early season the higher variability of wACWR and the lower variability in different wellness categories (i.e., wTM, wTS, wDOMS, wStress and wHI) were identified, which seem to suggest that wACWR did not negatively influence motivation and well-being status. This fact can be verified by the values assumed on the wACWR overall season that were lower than 1.5, values which in the literature seem to be associated with an increased injury rate [45]. On the other hand, the higher variability of wTM and wTS observed in the mid-season may be justified by the fact that players prefer higher loads to feel motivated. Moreover, this fact was strengthened by the evidence that in the mid-season a higher reduction in wCW (lower variability) was showed. Actually, this fact is defended in the literature, where it has been assumed that a higher TS suggests greater acute loads imposed with small-week variations [46].

Considering the overall season of the present study, a very large association was observed between wAW, wCW, wTS, and wDOMS measurement during the early season. Additionally, a large effect was also reported between wAW and wTS with wDOMS variable at the end-of-season. The greatest effect between well-being categories with wAW and wCW was coincident with earlier studies in team sports [47] and in an elite volleyball team [38], which reported a very large effect with wDOMS, wSleep, and wFatigue in all well-being categories, however in a different season period (i.e., mid-season). This discrepancy may be explained by the age of the participants, and by the difference in training load that players experienced during the pre-season [48]. Nevertheless, these relationships did not always report the same magnitude. For example, Mendes et al. [49] indicated a small association between workload and wDOMS in the same period of the season (i.e., early season). In the current study, a large association between wAW, wCW, and wTS during the early season with wHI category was verified. This fact is justified by the specific phase of periodization, where high training volume and fitness components development were registered, resulting in a greater perception of fatigue, muscle soreness, and a lower sleep quality. This is the second study which carefully analyzed the training load and well-being in young soccer players throughout an overall season (pre-season to the end-of-season, 36 weeks). However, there are some limitations that should be considered. Firstly, the number of included athletes was rather low. Nevertheless, a priori power analysis was conducted, and a sufficient statistical power was obtained through the size of the sample already mentioned. Secondly, only one team was considered in the study, which may limit the generalizability of our findings. Nevertheless, this issue is considered to be a regular limitation of longitudinal studies in elite contexts. In further research, it would be interesting to replicate the present study with more teams in the same season, level of competition, or even with different age groups. Furthermore, it would be pertinent to include female soccer players and different age categories in order to increase the generalizability of the reported results.

## 5. Conclusions

The aim of this study was to analyze the correlations between workload (AW, CW, and ACWR), TM, and TS with well-being variables in different periods of the season. Results showed that more variability in wAW happened in the end-of-season, it was harmonious with a higher variability of fatigue, stress, and HI. At the mid-season, the highest variation in wSleep was observed when both AW and CW showed lower variability. The mid-season was the period when TM and TS had higher variability. The ACWR generated lower variability in mostly wellness variables (i.e., wTM, wTS, wDOMS, wStress, and wHI). Moreover, there are a meaningful and relevant associations with training load (i.e., AW, CW, and TS) and well-being categories (i.e., HI, stress, sleep quality, and fatigue). Sport coaches and strength conditioning experts should be cautious on the significance that workload (acute or chronic) has on the wellness perceived responses of the players. Moreover, there should be an attempt to reduce the consequences of abrupt workload variations, which may compromise the performance, such as DOMS or fatigue. Stronger associations between workload and well-being variables of young soccer players in the early season were also disclosed. Thus, a greater consideration should be applied to better control the training process and maximize the development of young players. Thereby, wellness status may be considered as a useful tool to provide determinant subjective players’ information to the coaches, and thus possibly identifying important variations in training responses.

## Figures and Tables

**Table 1:** Monitoring during the full season.

Year	2019										2020
Months	May	June	July	Aug	Aug	Sept	Oct	Nov	Dec	Dec	Jan
Weeks	1–4	5–8	9–12	13	14–16	17–20	21–24	25–28	29–31	32	33–36
TS	20	23	19	4	15	21	20	18	14	5	20
Phase	First PP		Regional League							Second PP	Best of Iran (National)
Periods	Pre-Season		Early Season		Mid-Season					End-of-Season	
OG	-	-	-	-	3	3	4	5	3	-	8
NOG	2	3	3	-	-	-	-	-	-	-	-

TS, training session; PP, Preparation phase; OG, Official Games Number; NOG, Non-Official Games Number; W, week.

## Data Availability

The datasets used and/or analyzed during the current study are available from the corresponding author on reasonable request.
